# Supplementary material for: Implementation of Practical Surface SARS-CoV-2 Surveillance in School Settings
Source: mSystems. 2022 Jun 15;7(4):e00103-22. doi: 10.1128/msystems.00103-22 (PMC9426517; doi:10.1128/msystems.00103-22)
Supplement: TABLE S1 [file msystems.00103-22-st001.docx]

| Swabbing Medium (Extraction Facility) | | 0.5% SDS (UCSD) | | | | VTM (County) | | | |  |
| --- | --- | --- | --- | --- | --- | --- | --- | --- | --- | --- |
| RT-qPCR Facility | | UCSD | | County | | UCSD | | County | |  |
|  |  |  |  |  |  |  |  |  |  |  |
|  | | Avg Cq | # of Hits (out of 3) | Avg Cq | # of Hits (out of 2) | Avg Cq | # of Hits (out of 3) | Avg Cq | # of Hits (out of 2) |  |
| **A** | bathroom sink | 25.41 | 3 | 28.67 | 2 | 29.50 | 3 | 30.71 | 2 |  |
|  | toilet floor | 30.96 | 3 | 37.19 | 1 | 33.68 | 3 | 37.54 | 1 |  |
|  | bed headboard | 29.47 | 2 | Not Det. | 0 | Not Det. | 0 | Not Det. | 0 |  |
|  | bedside floor | 32.53 | 3 | Not Det. | 0 | 33.32 | 3 | Not Det. | 0 |  |
|  | bedside table | 31.31 | 3 | 34.05 | 2 | 29.69 | 3 | 30.98 | 2 |  |
|  | kitchen sink handles | 32.31 | 3 | 37.85 | 2 | 33.90 | 3 | 37.47 | 2 |  |
|  | kitchen table | 33.92 | 3 | 39.23 | 1 | 32.26 | 3 | 33.61 | 2 |  |
|  | desk top | 30.68 | 3 | 36.10 | 2 | 34.11 | 2 | 37.91 | 2 |  |
|  | desk chair | 30.26 | 3 | Not Det. | 0 | Not Det. | 0 | Not Det. | 0 |  |
|  | light switch | 28.06 | 3 | 37.92 | 1 | Not Det. | 0 | 41.24 | 1 |  |
| **B** | bathroom sink | Not Det. | 0 | Not Det. | 0 | Not Det. | 0 | Not Det. | 0 |  |
|  | bathroom door handle | Not Det. | 0 | Not Det. | 0 | Not Det. | 0 | Not Det. | 0 |  |
|  | toilet floor | Not Det. | 0 | Not Det. | 0 | Not Det. | 0 | Not Det. | 0 |  |
|  | bed headboard | Not Det. | 0 | Not Det. | 0 | Not Det. | 0 | Not Det. | 0 |  |
|  | bedside floor | Not Det. | 0 | Not Det. | 0 | Not Det. | 0 | Not Det. | 0 |  |
|  | bedside table | Not Det. | 0 | Not Det. | 0 | Not Det. | 0 | Not Det. | 0 |  |
|  | kitchen counter | Not Det. | 0 | Not Det. | 0 | Not Det. | 0 | Not Det. | 0 |  |
|  | kitchen sink handles | Not Det. | 0 | Not Det. | 0 | Not Det. | 0 | Not Det. | 0 |  |
|  | coffee table | 34.01 | 1 | Not Det. | 0 | Not Det. | 0 | Not Det. | 0 |  |
|  | light switch | Not Det. | 0 | Not Det. | 0 | 35.42 | 1 | 38.74 | 2 |  |
| **C** | bathroom sink | 32.14 | 2 | 38.4 | 1 | 33.66 | 1 | 39.01 | 2 |  |
|  | toilet floor | 33.75 | 2 | Not Det. | 0 | Not Det. | 0 | 36.78 | 2 |  |
|  | bed headboard | 33.68 | 2 | 39.36 | 1 | Not Det. | 0 | Not Det. | 0 |  |
|  | bedside floor | Not Det. | 0 | Not Det. | 0 | Not Det. | 0 | 37.91 | 1 |  |
|  | bedside table | 31.71 | 3 | 39.4 | 1 | 31.41 | 3 | 32.64 | 2 |  |
|  | kitchen counter | 32.36 | 3 | Not Det. | 0 | Not Det. | 0 | 38.44 | 2 |  |
|  | kitchen sink handles | 31.11 | 3 | 38.755 | 2 | 33.13 | 2 | 39.38 | 1 |  |
|  | kitchen table | 31.41 | 3 | 38.47 | 1 | Not Det. | 0 | 39.61 | 1 |  |
|  | desk top | 30.67 | 3 | 37.035 | 2 | 31.35 | 3 | 32.35 | 2 |  |
|  | light switch | 33.23 | 3 | Not Det. | 0 | Not Det. | 0 | Not Det. | 0 |  |
