# Supplementary material for: Implementation of Practical Surface SARS-CoV-2 Surveillance in School Settings
Source: mSystems. 2022 Jun 15;7(4):e00103-22. doi: 10.1128/msystems.00103-22 (PMC9426517; doi:10.1128/msystems.00103-22)
Supplement: TABLE S2 [file msystems.00103-22-st002.docx]

| **Lab** | **Sample collection medium** | **RNA extraction** | **RT-qPCR** |
| --- | --- | --- | --- |
| UCSD | SDS | Thermo pipeline | Thermo pipeline |
| SDPHL | VTM | PerkinElmer pipeline | PerkinElmer pipeline |
