# Supplementary material for: Implementation of Practical Surface SARS-CoV-2 Surveillance in School Settings
Source: mSystems. 2022 Jun 15;7(4):e00103-22. doi: 10.1128/msystems.00103-22 (PMC9426517; doi:10.1128/msystems.00103-22)
Supplement: TABLE S3 [file msystems.00103-22-st003.docx]

|  | | **Extraction Pipeline** | |
| --- | --- | --- | --- |
|  |  | Thermo (UCSD) | PerkinElmer (PHL) |
| **RT-qPCR Pipeline** | Thermo (UCSD) | Thermo | PE/Thermo |
|  | PerkinElmer (PHL) | PE/Thermo | PE |
